# CentroidAlign-Web: A Fast and Accurate Multiple Aligner for Long Non-Coding RNAs

**DOI:** 10.3390/ijms14036144

**Published:** 2013-03-18

**Authors:** Haruka Yonemoto, Kiyoshi Asai, Michiaki Hamada

**Affiliations:** 1 Department of Computational Biology, Graduate School of Frontier Sciences, the University of Tokyo, 5-1-5 Kashiwanoha, Kashiwa-shi, Chiba 277-8561, Japan; E-Mails: yonemoto haruka@cb.k.u-tokyo.ac.jp (H.Y.); asai@k.u-tokyo.ac.jp (K.A.); 2 Computational Biology Research Center (CBRC), the National Institute of Advanced Industrial Science and Technology (AIST), Tokyo Waterfront Bio-IT Research Building, 2-4-7 Aomi, Koto-ku, Tokyo 135-0064, Japan

**Keywords:** ncRNAs, rRNAs, multiple sequence alignment (MSA), structural alignment, secondary structure, consensus structures, web server

## Abstract

Due to the recent discovery of non-coding RNAs (ncRNAs), multiple sequence alignment (MSA) of those long RNA sequences is becoming increasingly important for classifying and determining the functional motifs in RNAs. However, not only primary (nucleotide) sequences, but also secondary structures of ncRNAs are closely related to their function and are conserved evolutionarily. Hence, information about secondary structures should be considered in the sequence alignment of ncRNAs. Yet, in general, a huge computational time is required in order to compute MSAs, taking secondary structure information into account. In this paper, we describe a fast and accurate web server, called CentroidAlign-Web, which can handle long RNA sequences. The web server also appropriately incorporates information about known secondary structures into MSAs. Computational experiments indicate that our web server is fast and accurate enough to handle long RNA sequences. CentroidAlign-Web is freely available from http://centroidalign.ncrna.org/.

## 1. Introduction

Various non-coding RNAs (ncRNAs), especially long non-coding RNAs (lncRNAs/lincRNAs) [1], are emerging as new players in molecular biology, demonstrating potential roles in the mechanism of diseases, such as cancers [2]. In the ENCODE project, the number of ncRNAs, including lncRNAs, reported is more than 6,000 [3], and this is one of the most important research themes in the project. When analyzing the evolution and functions of ncRNAs, multiple sequence alignment (MSA) is an important first step. It is known that the secondary structures of many ncRNAs are strongly related to their functions, and so, not only primary (nucleotide) sequences, but also secondary structures of ncRNAs are evolutionarily conserved [2]; Hence, it is important to consider secondary structure explicitly when aligning RNA sequences. However, the computational cost of aligning RNA sequences, while considering secondary structures, is huge: the computational cost of the aligning of two RNA sequences is *O*(*L*^6^), where *L* is the length of RNA sequences (see [4]).

Currently, there are several web servers that can be used for aligning multiple RNA sequences and that consider secondary structures: PicXAA-Web [5]; R-coffee [6]; LocARNA [7]; FoldAlign (for aligning two sequences) [8]; StrAl Webservice [9]; MAFFT [10]; Dynalign [11], and so forth. However, due to the high computational demands of aligning RNA sequences while considering secondary structures, most existing web servers cannot handle long RNA sequences (e.g., rRNAs [12] or lincRNAs [1,13]).

We have developed a novel web server (called “CentroidAlign-Web”) for aligning multiple RNA sequences by extending CentroidAlign [14], which is a fast and accurate multiple aligner for RNA sequences that considers secondary structures. The features of CentroidAlign-Web are summarized as follows:

- CentroidAlign-Web can accept long RNA sequences, such as rRNAs. In order to handle those RNA sequences, we have reduced the time complexity of CentroidAlign by integrating the Rfold algorithm [15] into it (see the next section for details).
- Users can (optionally) give the secondary structure(s) of input sequences, if this information is available. For example, secondary structures of long RNA sequences from HIV-1 [16], HCV (hepatitis C virus) [17] and lincRNA (the steroid receptor RNA activator (SRA)) [18] have been recently determined by combining experimental techniques with computational approaches. This secondary structure information is useful for estimating multiple alignments.
- CentroidAlign-Web has an interface in which users can specify a region of the human genome (hg18) from which to extract a multiple alignment, and re-align that region using CentroidAlign. Because recent studies have suggested that re-alignment of genome sequence alignments reveals new non-coding RNAs [19], this function will be useful.

Computational experiments conducted in this study indicate that our web server is fast enough to compute a multiple alignment for long RNA sequences, and known secondary structure information can improve multiple alignments of RNA sequences. CentroidAlign-Web is freely available from http://centroidalign.ncrna.org/, and will be useful for research on non-coding RNAs.

## 2. Materials and Methods

### 2.1. CentroidAlign

**Figure A1:**
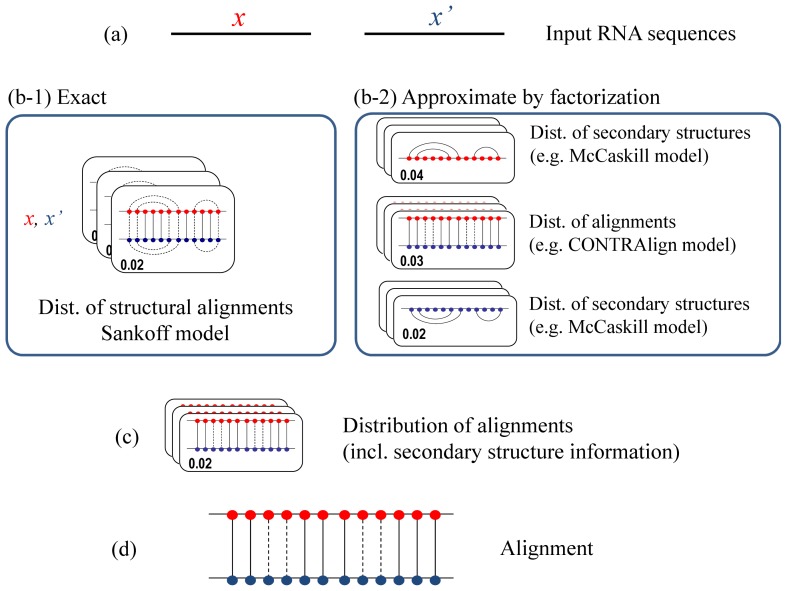
Overview of the pairwise alignment step in the CentroidAlign algorithm [14]. (**a**) The input is two RNA sequences, (*x; x*^′^), to be aligned; (**b-1**) The exact algorithm of CentroidAlign considers a probability distribution of structural alignments between *x* and *x*^′^, which gives simultaneously the alignments between nucleotides and those between base-pairs (e.g., Sankoff model [4]); (**b-2**) The exact case can be approximated by factorizing the distribution of structural alignments into (i) a distribution of secondary structures of *x* (e.g., the CONTRAfold [27] or McCaskill [22] models); (ii) a distribution of pairwise alignments between *x* and *x*^′^ (e.g., the CONTRAlign model [23]); and (iii) a distribution of secondary structures of *x*^′^; (**c**) By marginalization of the distribution(s) in (b), we obtain a distribution of alignments (*) in which the information about secondary structures is included; (**d**) The best multiple alignment is estimated based on maximizing expected accuracy (MEA) [24] in which the SPS scores of predicted alignments are optimized with respect to the distribution (*) of pairwise alignments given in (c). It should be emphasized that the computational cost of the exact algorithm is ≈ *O*(*L*^6^), while it is reduced to ≈ *O*(*L*^3^) in the approximate algorithm, where *L* is the (maximum) length of two input sequences.

CentroidAlign [14] is a fast and accurate aligner for multiple RNA sequences. In contrast to usual MSA tools for DNA/protein sequences (e.g., ClustalW [20] or ProbCons [21]), CentroidAlign can consider (common) secondary structures among input RNA sequences when aligning RNA sequences (cf. Figure A1). Because secondary structures of RNAs are often conserved in their evolution, it is important to consider secondary structures in multiple alignments of RNA sequences. However, considering a common secondary structure in a multiple alignment (this kind of alignment is often called “structural” alignment) entails a huge computational cost (cf. [4]). CentroidAlign reduces the computational costs by several heuristic techniques, factorizing a probability distribution of structural alignments (given by, e.g., the Sankoff model [4]) into (i) a probability distribution of secondary structures (given by, e.g., the McCaskill model [22]) and (ii) a probability distribution of (usual) alignments (given by, e.g., the ProbCons model [21]) (b-2 in Figure A1). This approximation leads to an algorithm based on a base-pairing probability matrix (BPPM) for each RNA sequence ( a BPPM gives the (marginal) probability of every base-pair with respect to a probability distribution of secondary structures) and an aligned-base probability matrix (ABPM) for every pair of RNA sequences (an ABPM gives the (marginal) probability of every aligned base-pair with respect to a probability distribution of alignments). Both matrices include information about the ambiguity of secondary structures and alignments. The result is that the time complexity of the pairwise alignment step in CentroidAlign is *O*(*L*^3^ + *c*^2^*dL*^2^) ≈ *O*(*L*^3^), where *L* is the length of input sequences and both *c* and *d* are constants independent of *L*.

Moreover, we have integrated the probabilistic consistency transformation (PCT) of the alignment probability matrix [21] into the proposed estimator. Finally, the extension to multiple alignment is conducted by a progressive alignment algorithm similar to CONTRAlign [23].

Note that CentroidAlign employs an estimator based on maximum expected accuracy (MEA), which has been successfully applied in much software in the field of bioinformatics; see the review by Hamada and Asai [24] for details. In CentroidAlign, the sum-of-pair scores (SPS) [25] is optimized for predicting multiple alignments of RNA sequences (cf. c and d in Figure A1).

### 2.2. Rfold

The Rfold algorithm, which was proposed in [15], computes a BPPM for a given RNA sequence. In the computation of the BPPM, Rfold can use the maximum distance of base-pairs in a predicted secondary structure, which enables it to handle longer RNA sequences. The time complexity of Rfold is *O*(*w*^2^*L*), where *w* is the maximum size (span) of base-pairs, while the time complexity of algorithms that compute a full BPPM (such as the McCaskill algorithm [22]) is *O*(*L*^3^), where *L* is the length of the RNA sequence.

### 2.3. Dataset Utilized in Computational Experiments

Table 1 shows a summary of the dataset used in this study. RNA families whose length is more than 800 are taken from seed alignments in the Rfam 11.0 database (August 2012) [2]. Note that those seed alignments give high-quality benchmark datasets, because they are manually curated MSAs, which take into consideration (consensus) secondary structures.

## 3. Results and Discussion

### 3.1. CentroidAlign Web Application (CentroidAlign-Web)

**Figure 1:**
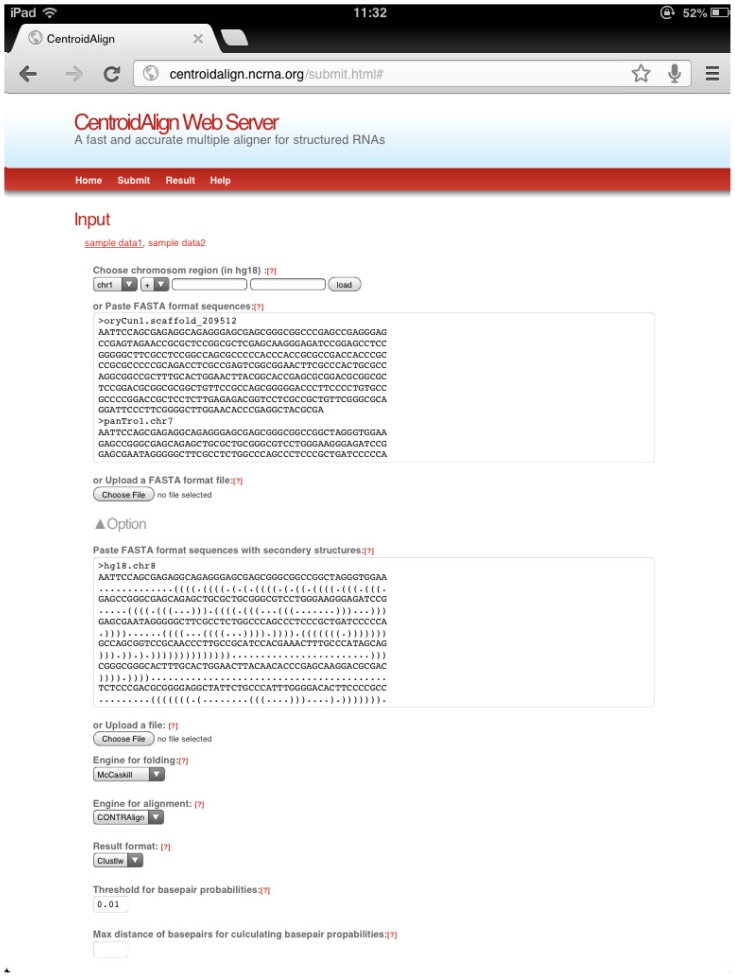
Input page of CentroidAlign-Web, in which RNA sequences are given in the FASTA format. Additionally, by using the interface: (i) users can give secondary structures for parts of input RNA sequences; (ii) users can specify a region of the human genome (hg18); (iii) users can utilize the Rfold algorithm to compute base-pairing probability matrices (BPPMs), with a user-given maximum distance for base-pairs.

**Figure 2:**
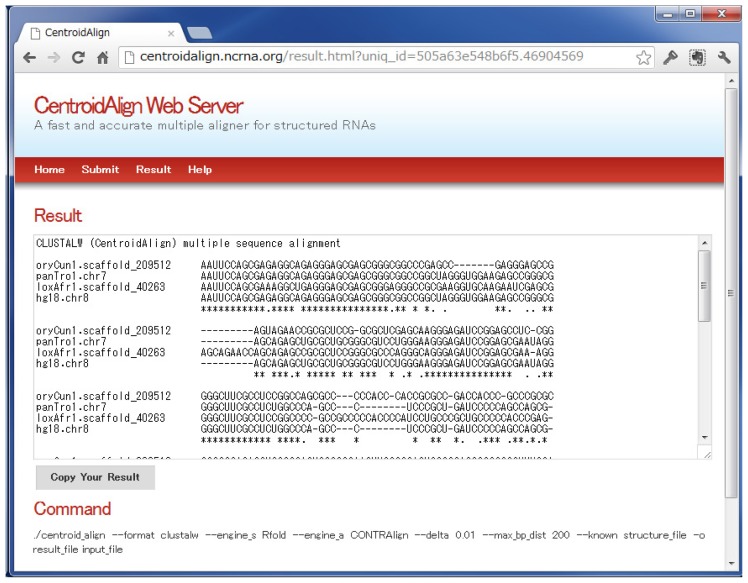
Results page of CentroidAlign-Web. The output of an multiple sequence alignment (MSA) is in either ClustalW format or multi-FASTA format. The complete command line information is also provided on the results page. Users can copy the result to the clipboard for use in the next analysis: e.g., common secondary structure prediction using CentroidAlifold [26] (http://www.ncrna.org/centroidfold) with the predicted multiple alignment from CentroidAlign-Web.

**Figure 3:**
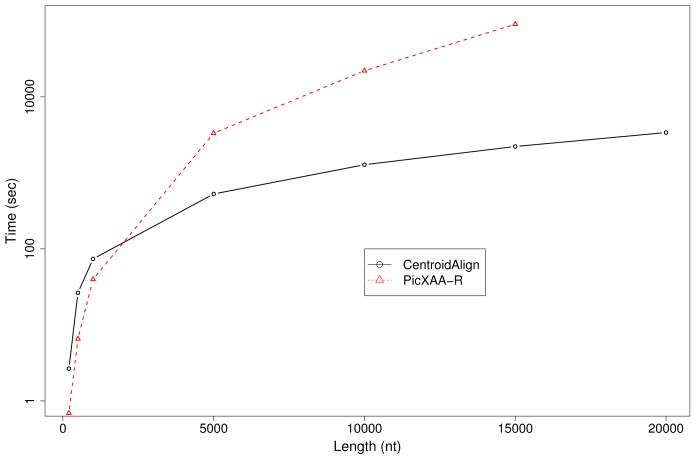
Computational time of CentroidAlign and PicXAA-R for RNA sequences with various length. In this experiment, five random RNA sequences were utilized for each dataset. The black and red lines correspond to CentroidAlign and PicXAA(-R), respectively. PicXAA did not finish within three days for the length of 20,000 nt.

Usage of the server is quite simple. Users can paste sequences in FASTA format (http://www.ebi.ac.uk/help/formats.html#fasta) into a text area or upload a FASTA file, then click on the “submit” button (Figure 1). The server responds with a multiple alignment (Figure 2)(See Table 2 and Figure 3 for computational time of our web server). The resulting format is multiple alignment format (MAF) or clustalW. By expanding “Options” in the interface, users can adjust several internal parameters of the web server (see Table 3 for the detailed parameters). There are three major advantages in this web server. (1) The maximum distance between the two bases of a base-pair can be specified (by users) in order to reduce computational cost for computing BPPMs (which is the most time-consuming part of CentroidAlign) (see Section 3.1.1.). This option ensures that the alignment finishes in a practical amount of time, even if users’ query sequences are relatively long (e.g., rRNAs); (2) Users can utilize secondary structural information for alignment. An example of the required format is given on the help page (http://centroidalign.ncrna.org/help.html). When the structures of users’ query sequences are experimentally determined, the probabilities of positions at which bases make a pair should be 1 and, otherwise, 0 (cf. Section 3.1.2.). Using actual (not predicted) probabilities should enable more accurate alignment of structured RNAs. (3) Users can extract an MAF region (from the hg18 17way MULTIZ alignment) by specifying chromosome, start position, end position and strand. The sequences in the multiple alignment are realigned by CentroidAlign.

When submitting the job, the user is given a “Job ID” and a link to the results (multiple FASTA format or ClustalW format). Users can retrieve the results by using the Job ID at a later time. Then, users can copy the result to the clipboard and use it in the next analysis, for example, in common secondary structure prediction of the multiple alignment, using CentroidAlifold [26] http://www.ncrna.org/centroidfold. Additionally, a complete set of command line options can be obtained, which is useful for users of the command line version of CentroidAlign.

#### 3.1.1. Incorporating the Rfold Algorithm into the Web Server

In CentroidAlign-Web, we incorporated the Rfold algorithm (cf. Section 2.2) to compute the BPPM for each RNA sequence in the input sequences. As a result, the total computational cost of CentroidAlign is reduced to *O*(*w*^2^*L*+*c*^2^*dL*^2^) ≈ *O*(*L*^2^), where *w* is the maximum length of base-pairs, *L* is the length of input sequences and both *c* and *d* are constants independent of *L* (Note that the computational cost of the original CentroidAlifold is ≈ *O*(*L*^3^); see Section 2.1). This reduction of computational cost enables the prediction of MSAs for longer RNA sequences (e.g., ribosomal RNA sequences or lincRNAs), taking into account information about secondary structures.

#### 3.1.2. BPPM for an RNA Sequence with a Secondary Structure

For an RNA sequence, *x*, with a (known) secondary structure, *y*, a BPPM for the sequence is given by:

$$p_{ij}=\{\begin{array}{ll} 1 & \text{if }x_{i}\;\text{and }x_{j}\;\text{form}\;\text{a base}-\text{pair in a given structure}\\ 0 & \text{otherwise} \end{array}$$

instead of utilizing the BPPM calculated, e.g., by the McCaskill algorithm [22]. In this way, we can seamlessly incorporate information about secondary structures into computing multiple alignments in CentroidAlign.

### 3.2. Computational Experiments

In our computational experiments, we focused on relatively long RNA sequences in order to show that our web server can handle longer sequences than existing software. (Note that there exists several studies that extensively evaluate among multiple aligners (including CentroidAlign) for short (~ 500 nt) RNA sequences with low sequence similarities [14,28], indicating that CentroidAlign achieved good performance to those datasets.) We have tested six RNA families (from the Rfam 11.0 database [2]), whose average length is relatively long (from 700 to 1,800 bases). The largest dataset contains 84 RNA sequences, whose average length is around 1,800. See Table 1 for the details of the datasets. We conducted our computational experiments on a Linux machine with a 3.33 GHz Intel(R) Xeon(R) CPU W5590 processor and 32 GByte of memory. Note that the current version of CentroidAlign-Web is also implemented on a machine with the same specification.

The results are shown in column (a) in Table 2. In the experiments, Rfold with the maximum size of base-pairs set to be 300 was employed for the probability distribution of secondary structures (in other words, Rfold was employed to calculate the base-pairing probability matrix of secondary structures, setting the maximum distance between base-pairs to 300 nt); CONTRAlign was utilized for the probability distribution of pairwise alignments. The computational time for the largest dataset (RF01960, which contains 84 sequences with an average length of 1,791) is a few hours; for a moderately sized dataset (RF01959, which contains 19 sequences with an average length of 1,190), the computational time is less than 300 s. We compared CentroidAlign with PicXAA(-R) (version 1) [5,28], which is one of the fastest multiple aligners for RNA sequences, wherein the information of secondary structures is taken into account. For a larger dataset (e.g., RF01960 and RF00177), CentroidAlign was faster than PicXAA, and SPSs of CentroidAlign were consistently better than those of PicXAA among all dataset (Table 2). In addition, in Figure 3, we show the computational time of multiple sequence alignment for five (random) sequences with various lengths up to 20,000. This result indicated that, for longer RNA sequences, CentroidAlign is much faster than PicXAA (e.g., CentroidAlign took 40 minutes for five sequences of 15,000 nt, while PicXAA took more than 1 day), which is one of the advantages of our web sever.

Finally, in order to examine whether information about secondary structures improves the accuracy of MSAs of RNA sequences, we conducted computational experiments using known secondary structures. The ratio of known secondary structures in the input sequences was 25%, 50% or 75% (corresponding to columns (b), (c) and (d), respectively, in Table 2). The secondary structures are given by mapping consensus secondary structures (in seed alignments) to the RNA sequence. Table 2 shows that the information about secondary structures (slightly) improved the accuracy of multiple alignments, which indicates the usefulness of known secondary structures in MSAs. The use of secondary structure information seems to have more impact on datasets RF01272 and RF01825, which correspond to RNA families with lower primary sequence identity (see MPI values in Table 1), compared to datasets containing sequences with higher (*>*70%) identity (Table 2), indicating that the importance of secondary structures in RNA families with low sequence conservation.

### 3.3. Future Work

We are planning to incorporate biochemical experimental information (such as SHAPE) into the web server, because such information can be used to determine secondary structure [29] by employing a recently developed method that enables the updating of the BPPM according to experimental information [30].

Recent studies have clearly indicated the importance of lincRNAs [13]. Not only lincRNAs are longer than conventional non-coding RNAs (such as snoRNAs and miRNAs), but also most lincRNAs exhibit low sequence similarity. We therefore plan to apply ourWeb Server to the detailed analysis of lincRNAs (such as SRA [18] and HOTAIR [31]), which might lead to important biological findings.

## 4. Conclusions

In this paper, we have introduced CentroidAlign-Web, a web server for predicting multiple alignments of long RNA sequences. We showed that the web server is capable of dealing with long RNA sequences, such as rRNAs, and that information about secondary structures can be used to improve the accuracy of multiple alignments. CentroidAlign-Web is freely available from http://centroidalign.ncrna.org/, which would be useful to researches of non-coding RNAs.

## Figures and Tables

**Table 1:** Datasets used in this study. Each family is taken from the Rfam 11.0 database [2]. “Num”, “Average length (nt)” and “MPI” mean the number of sequences in the family, the average length of sequences and the mean pairwise identity of sequences in each family, respectively.

Dataset name	Accession	Num	Average length(nt)	MPI(%)	Description
SSU rRNA eukarya	RF01960	84	1791.20	80.00	Eukaryotic small subunit ribosomal RNA
SSU rRNA bacteria	RF00177	93	1524.50	80.00	Bacterial small subunit ribosomal RNA
SSU rRNA archaea	RF01959	19	1480.50	81.00	Archaeal small subunit ribosomal RNA
Sacc telomerase	RF01050	13	1189.50	70.00	Saccharomyces telomerase
snR86	RF01272	5	998.40	69.00	Small nucleolar RNA snR86
RUF21	RF01825	5	691.80	65.00	RNA of unknown function 21

**Table 2:** Computational results for various ratios of known secondary structures: **(a)** no secondary structure information is given; **(b)** (resp. **(c)** and **(d)**) 25% (resp. 50% and 75%) of secondary structures for input sequences are given. The “SPS” columns show the sum-of-pairs-score of a predicted multiple alignment [25]. In CentroidAlign-Web, we utilized the Rfold model [15] (where the maximum size (span) of base-pairs is set to 300) for a model of RNA secondary structures; we utilized the CONTRAlign model [23] for a model of pairwise alignments (see Figure A1). In PicXAA-R, we conducted a standalone version of PicXAA-R (version 1.0) and the default parameters were utilized. A Linux OS machine with a 3.33 GHz Intel(R) Xeon(R) CPU W5590 processor and 32 GByte of memory was used in this experiment. See Table 1 for detailed information about the datasets used.

			CentroidAlign								PicXAA-R	
				
			(a) 0%		(b) 25%		(c) 50%		(d) 75%			
ID	Num	Average length (nt)	SPS	Time(s)	SPS	Time(s)	SPS	Time(s)	SPS	Time(s)	SPS	Time(s)
RF01960	84	1791.2	0.9173	6546.08	0.9164	5924.78	0.9179	5317.99	0.9208	4687.98	0.9121	8945.82
RF00177	93	1524.5	0.9560	5874.18	0.9576	5314.39	0.9589	4782.40	0.9572	4228.57	0.9548	7544.52
RF01959	19	1480.5	0.9800	569.66	0.9816	474.21	0.9817	364.89	0.9821	251.17	0.9786	508.96
RF01050	13	1189.5	0.8848	273.87	0.8840	219.46	0.8861	165.06	0.8926	111.81	0.8764	155.42
RF01272	5	998.4	0.8975	80.06	0.9049	64.75	0.9152	48.97	0.9286	34.31	0.8913	35.58
RF01825	5	691.8	0.8319	44.80	0.8467	36.38	0.8261	27.73	0.8572	18.98	0.8236	12.53

**Table 3:** Adjustable parameters in CentroidAlign-Web. Each parameter can be altered in the “Options” control.

Parameter name	Description	Possible	Default
Engine for folding	Probabilistic model of secondary structures	McCaskill, CONTRAfold, Rfold 1	Rfold
Engine for alignment	Probabilistic model of pairwise sequence alignments	CONTRAlign, ProbCons 2	CONTRAlign
Result format	Output format	ClustalW, MFA	ClustalW
Threshold for base-pair probabilities	Threshold for base-pairing probabilities	0 to 1	0.01
Max distance of base-pairs	The maximum distance of base-pairs	More than 0	300

1 CONTRAfold and McCaskill are probability distributions of secondary structures of RNA sequences proposed in [22,27], respectively;

2 CONTRAlign and ProbCons are probability distributions of pairwise alignments proposed in [21,23], respectively;

3 If the length of RNA sequences is long, users should specify this value in order to reduce the computational cost.
